# Reliable approaches to extract high-integrity RNA from skin and other pertinent tissues used in pain research

**DOI:** 10.1097/PR9.0000000000000818

**Published:** 2020-03-27

**Authors:** Peter M. LoCoco, Jacob T. Boyd, Claudia M. Espitia Olaya, Ashley R. Furr, Dawn K. Garcia, Korri S. Weldon, Yi Zou, Erin Locke, Alejandro Tobon, Zhao Lai, Shivani B. Ruparel, Nikita B. Ruparel, Kenneth M. Hargreaves

**Affiliations:** a Department of Endodontics, University of Texas Health Science Center at San Antonio, San Antonio, TX, USA; b University of Arizona Cancer Center, Tucson, AZ, USA; c Greehey Children's Cancer Research Institute, University of Texas Health Science Center at San Antonio, San Antonio, TX, USA; d Department of Neurology, South Texas Veterans Health Care System, San Antonio, TX, USA; e Department of Molecular Medicine, University of Texas Health Science Center at San Antonio, San Antonio, TX, USA

**Keywords:** RNA-sequencing, RNA extraction, Skin, RNA integrity assessment, RIN assignment, Cryosectioning

## Abstract

**Introduction:**

Comprehensive mRNA sequencing is a powerful tool for conducting unbiased, quantitative differential gene expression analysis. However, the reliability of these data is contingent on the extraction of high-quality RNA from samples. Preserving RNA integrity during extraction can be problematic, especially in tissues such as skin with dense, connective matrices and elevated ribonuclease expression. This is a major barrier to understanding the influences of altered gene expression in many preclinical pain models and clinical pain disorders where skin is the site of tissue injury.

**Objective:**

This study developed and evaluated extraction protocols for skin and other tissues to maximize recovery of high-integrity RNA needed for quantitative mRNA sequencing.

**Methods:**

Rodent and human tissue samples underwent one of the several different protocols that combined either RNA-stabilizing solution or snap-freezing with bead milling or cryosectioning. Indices of RNA integrity and purity were assessed for all samples.

**Results:**

Extraction of high-integrity RNA is highly dependent on the methods used. Bead-milling skin collected in RNA-stabilizing solution resulted in extensive RNA degradation. Snap-freezing in liquid nitrogen was required for skin and highly preferable for other tissues. Skin also required cryosectioning to achieve effective penetration of RNA-stabilizing solution to preserve RNA integrity, whereas bead milling could be used instead with other tissues. Each method was reproducible across multiple experimenters. Electrophoretic anomalies that skewed RNA integrity value assignment required manual correction and often resulted in score reduction.

**Conclusion:**

To achieve the potential of quantitative differential gene expression analysis requires verification of tissue-dependent extraction methods that yield high-integrity RNA.

## 1. Introduction

Comprehensive, unbiased RNA sequencing (RNA-seq) has become a widely used approach with more than 100 different methodological applications, the most common being differential gene expression (DGE) analysis, which determines quantitative expression changes that occur to the active transcriptome.^10,32,38^ In the field of pain research, one of the exciting applications of RNA-seq is to help guide the development of next-generation nonopioid-based analgesics through the identification of novel target genes in human-derived tissues.^46^ Recent studies have already shed light on the altered molecular landscapes of various tissues collected from chronic pain patients.^30,33,34^ Given its current impact, the Federal Pain Research Strategy has emphasized the continued use and development of RNA-seq to address top research priorities in the pain field.^28^ Yet despite its promise, caution is warranted as RNA-seq results easily can be confounded by methodological limitations. Thus, as the reliance on RNA-seq increases, it is critical that pain researchers meticulously validate their methods to maximize data set reliability.

Importantly, the accuracy, and thus reliability, of the DGE analyses is largely dependent on sample RNA integrity. Sample mRNA must be highly intact to accurately reflect ongoing gene expression; otherwise, degradation bias will increase intragroup and intergroup variances and ultimately false discovery rates.^9^ RNA degradation can have striking effects on the identification of true differentially expressed genes, with one study citing 4% and 26% of genes changing as RNA integrity values shifted from 9.3 to 7.9 and 6.2, respectively.^15^ Transcripts also are susceptible to differential degradation in a tissue-specific manner, which can further confound the reliability of DGE analyses.^15,20^ Therefore, it is imperative for experimenters to rigorously validate their RNA extraction procedures to minimize the confounding influence of degradation bias.

The extraction of high-integrity RNA from samples for sequencing experiments has become more routine, notably since the advent of RNA-stabilizing solutions and better homogenization strategies.^7,27^ Still, certain tissues are considerably more challenging to extract than others. One of the particular importance to the pain field is skin, because of its high endogenous expression of ribonucleases and its densely fibrous, yet tensile nature.^40^ Although several methods have been reported for skin,^5,6,8,12,14,43^ RNA integrity remains a primary issue. Given that paw skin is used in many inflammatory and neuropathic pain models, validation of a robust extraction protocol is important. A reliable extraction method for skin would certainly benefit future transcriptomic studies, especially those involving human biopsies. Therefore, the purpose of this study was to develop a reliable, reproducible extraction protocol that preserves high-integrity RNA from skin. Using indices for RNA yield, purity, and integrity, we evaluated results obtained from human and rodent skin biopsies as well as several other tissue types commonly used in pain research, including dorsal root ganglia (DRG), trigeminal ganglia (TG), spinal cord, and muscle. Collectively, this work emphasizes the critical importance of verifying extraction methods before proceeding forward with quantitative mRNA-seq.

## 2. Materials and methods

### 2.1. Human subjects

Regulatory approval was obtained from the Human Subjects Institutional Review Board at the University of Texas Health Science Center at San Antonio (#20160027HU). Informed consent was obtained from patients according to protocol guidelines set by the Institutional Review Board. Samples were from both male and female participants of older than 55 years. The study was performed in accordance with the Declaration of Helsinki.

### 2.2. Human skin biopsy collection

Skin punch biopsies were taken 10-cm proximal to the lateral malleolus. Marked biopsy sites were cleaned with alcohol swabs and then injected with 2% lidocaine/epinephrine (Hospira, Lake Forest, IL) solution to anesthetize the skin. Single-use circular punches (4 mm, Sklar Instruments) were used with rotational motion and slight pressure to biopsy to a depth of 4 mm. The edge of each biopsy was lifted with forceps to remove the underlying fatty tissue. Biopsy sites were covered with pressure bandage for 24 hours. Biopsies were collected in sterile 2-mL cryotubes and preserved by (1) immersion in ice-cold RNA*later* (Qiagen, Hilden, Germany) for 4 to 6 hours, or (2) immediate snap freezing on liquid nitrogen. Cryotubes were stored at −80°C until processing.

### 2.3. Animals

Male Sprague–Dawley rats (Crl: SD; Charles River, Wilmington, MA) aged 20 weeks, male and female C57BL/6J mice aged 8 to 20 weeks (Jackson), male BKS.Cg-Dock7^m^+/+Lepr^db^/J aged 16 weeks (BKS.Lepr^db/+^, Jackson),^17^ male athymic nude mice aged 18 weeks (Fox1^nu^; Envigo, Indianapolis, IN), and male BALB/cJ mice aged 12 weeks (Jackson) were used in this study. Rats were housed in groups of 3 and mice in groups of 4 to 5. Animals were maintained on a 12-hour light–dark cycle with ambient temperatures between 20 and 22°C, with food and water freely available. Animal protocols were approved by the Institutional Animal Care and Use Committee of the University of Texas Health Science Center at San Antonio and conformed to International Association for the Study of Pain and federal guidelines.

### 2.4. Rodent tissue collection

At sacrifice, the following tissues were collected: glabrous hindpaw skin, lumbar DRG, TG, spinal cord, tongue, incisor apical papilla, and/or masseter muscle. Skin biopsies (4 mm) were always performed first, within 60 seconds of sacrifice and either collected into RNA*later* or in empty cryotubes that were immediately snap frozen. Other tissues of interest were then dissected as quickly as possible and either immersed in RNA*later* or snap frozen. All tissues were stored at −80°C until processing.

### 2.5. RNA extraction

Specimens underwent extraction according to one of the following protocols:

#### 2.5.1. RNAlater + Bead Ruptor

Samples in RNA*later* were first thawed on ice. Meanwhile, the processing chamber of a Bead Ruptor 24 (Omni International, Kennesaw, GA) was precooled to 0°C with the BR-Cryo Cooling Unit (Omni). Reinforced screw-cap microtubes (2-mL) with 2.8-mm ceramic beads (Omni) were filled with 900 μL QIAzol reagent (Qiagen). Thawed samples were transferred to QIAzol-filled bead tubes then placed on the tube carriage. Samples were homogenized for 40 seconds at 7.10 m/s and then placed on ice for 5 minutes. For complete homogenization, DRG and TG required one cycle, spinal cord and teeth required 2 cycles, and skin required 3 cycles. Afterwards, samples were transferred to sterile 1.5-mL tubes and centrifuged for 3 minutes at 8000*g* to remove debris. Supernatants were collected into sterile 2-mL tubes on ice before RNA isolation/purification.

#### 2.5.2. Snap freeze + Bead Ruptor

Previously snap-frozen tissues were placed on dry ice from cryostorage. Bead-filled tubes and the Bead Ruptor were then prepared as above. Samples were homogenized using either the 1-, 2-, or 3-cycle bead-mill protocol described above. Homogenates were centrifuged to remove debris and then transferred to 2-mL tubes on ice before RNA purification.

#### 2.5.3. Snap-freeze + TissueLyser LT

Snap-frozen tongue samples were homogenized with a different bead-milling protocol. First, 2-mL tubes were filled with 1-mL QIAzol and a 7-mm stainless steel ball bearing (Qiagen). Tongue samples were transferred to QIAzol-filled tubes and loaded onto a 12-tube adapter (Qiagen) for the TissueLyser LT bead-mill (Qiagen). Samples were milled at 50 Hz for 45 seconds, placed on ice for 5 minutes, and then repeated to ensure complete homogenization. Homogenates were centrifuged to remove debris and transferred to sterile 2-mL tubes on ice before RNA purification.

#### 2.5.4. Snap-freeze + cryosection

First, a 34°/80 mm MX35 Premier + cryostat blade (ThermoFisher, Waltham, MA), forceps, and aluminum cylinders (5-mm internal diameter) were sprayed with RNaseZap (ThermoFisher), wiped and then acclimated in a HM550 cryostat (Microm) held at −21°C. Samples were transferred from −80°C to the cryostat along with sterile 2-mL tubes. Aluminum cylinders were placed atop optimal cutting temperature (OCT)–coated chucks and filled with 60 μL RNase-free water. Once frozen, tissue specimens were positioned within the cylinder followed by another 60 μL RNase-free water and then quick-frozen with a 3-cm stainless steel heat extractor. Ice-encased samples were separated from the cylinder and mounted onto the chucks with OCT. Approximately 20 cryosections (16 μm) were cut and then carefully brushed into cold 2-mL tubes with precooled forceps. This was repeated until the entire sample was processed. Tubes were handled only by the open cap to minimize heat transfer. After cryosectioning, 1 mL QIAzol was quickly added followed by vortexing for 60 seconds. Samples were then placed on ice until RNA purification.

#### 2.5.5. RNAlater + cryosection

Samples in RNA*later* were thawed on ice and then processed according to the cryosectioning protocol described above. After vortexing, samples were placed on dry ice until RNA purification.

### 2.6. RNA isolation and purification

Extracts underwent isolation and purification using the RNeasy Plus Universal Mini Kit (Qiagen). Briefly, 100 μL gDNA eliminator solution and 180 μL chloroform were dispensed into extracts, with shaking after each. After 5 minutes, extracts were centrifuged at 4°C for 15 minutes at 14,000*g*. The upper aqueous phase (∼600 μL) from each sample was transferred to new 2-mL microtubes. After, 600 μL of 70% ethanol was added to extracts with vigorous mixing, transferred to spin columns, and then centrifuged at 12,000*g* for 30 seconds. Transfer and centrifugation were repeated, and then, the spin columns were washed once with 700 μL RWT buffer and twice with 500 μL RPE buffer. Spin columns were placed into 1.5-mL microtubes, and then 30 μL of RNase-free water was carefully applied to the membrane of each column followed by centrifugation at 14,000*g* for 60 seconds. Eluates were reapplied to their corresponding membrane and recentrifuged. RNA concentrations and purity were determined with a Nanodrop 2000 spectrophotometer (ThermoFisher).

### 2.7. RNA integrity assessment

Samples were delivered to the Genome Sequencing Facility at the Greehey Children's Cancer Research Institute for RNA integrity assessment. RNA integrity number (RIN) or RNA quality number were determined with a Bioanalyzer (Agilent, Santa Clara, CA) or Fragment Analyzer (Agilent), respectively. Concentrated samples (>50 ng/μL) were processed using regular/NanoChip kits (Agilent), whereas dilute samples (<50 ng/μL) required high-sensitivity/PicoChip kits (Agilent). High-integrity RNA was defined with RIN/RNA quality number values ≥ 7.0.^15,20,42^ ProSize 3.0 (Agilent) was used to manually correct misidentified rRNA peaks in electropherograms where degradation affected the fragment baseline. Identification of 18S (∼1.9 kb) and 28S (∼4.7 kb) peaks were checked first, and if incorrect, the user redesignated the appropriate region. Peak integration boundaries were then validated. Start- and end-points were manually repositioned to only bound the points meeting the fragment baseline. RNA integrity number values were then updated after correction.

### 2.8. Graphics

Modifications to images and electropherograms (for size) were made using OmniGraffle 7.11.3 (the Omni Group, Seattle, WA).

### 2.9. Statistical analysis

Group mean values were compared using Student's *t* test or one-way analysis of variance with Tukey's or Sidak's post hoc tests for pairwise comparisons, where appropriate. Pearson correlation coefficients were determined to establish the linear dependence between RIN values and 28S/18S rRNA ratios for all samples included in this study. Linear regression confirmed the correlation value and 95% confidence bands from the best-fit line. All statistical tests were two-sided with the significance threshold established with α = 0.05. Analyses were performed in GraphPad Prism 8.0. Multidimensional scaling plots were generated from selected samples with R v3.5.2.^41^

## 3. Results

### 3.1. RNA-stabilizing solution does not prevent RNA degradation in skin

**Figure 1.**
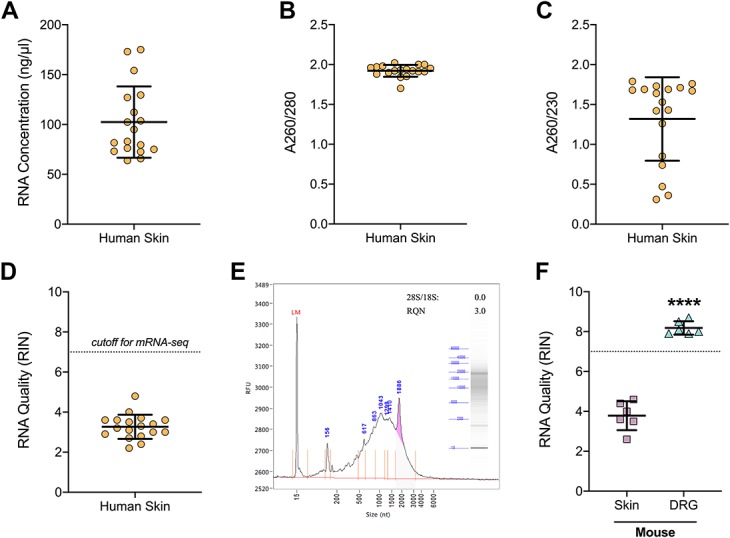
RNA-stabilizing solution does not prevent RNA degradation in bead-milled human or rodent skin. (A–D) Scatter plots of RNA concentrations (A), A260/280 values (B), A260/230 values (C), and RIN values (D) determined for extracted RNA from each patient skin biopsy (orange circles, n = 18 samples). (E) Representative electropherogram with associated RIN and 28S/18S rRNA values from a patient sample. (F) Scatter plot of RIN values for mouse glabrous skin (light purple circles) and lumbar DRG (teal squares) samples. The dotted line on RIN scatter plots denotes the cutoff value for sample RNA integrity to be considered for mRNA-seq and subsequent quantitative differential gene expression analysis. Black horizontal bars in each plot represent the calculated group mean ± SD. ****P < 0.0001 by two-tailed Student's *t*-test, n = 6 mice/group. DRG, dorsal root ganglia; RIN, RNA integrity number.

We sought an extraction protocol that would routinely generate high-integrity RNA from skin biopsies, which has proven extremely challenging given the high expression level of ribonucleases in skin.^11,40^ Successful preservation of RNA from human tissue samples, including skin, has been reported previously with the use of RNA*later*.^14,27^ Thus, our initial collection protocol included the use of RNA*later* to inhibit ribonucleases and preserve transcript integrity. Accordingly, patient skin biopsies were immersed in ice-cold RNA*later* for several hours upon collection followed by cryostorage. RNA was extracted using a 3-cycle bead-milling homogenization strategy in combination with silica membrane-based purification. This extraction protocol yielded high RNA concentrations (Fig. 1A) and high nucleic acid purity (Fig. 1B, C), although there was moderate variability in A260/230 values, likely due to residual salt accumulation.^26^ However, RIN values were poor for all 18 samples, averaging 3.3 ± 0.6 (Fig. 1D). Indeed, substantial degradation is apparent, as evidenced by the lack of ribosomal peaks and accumulation of small RNA fragments in the fast region of a representative electropherogram (Fig. 1E). To determine whether these results were unique to human skin, we performed the same extraction protocol using mouse glabrous skin biopsies. We also tested mouse lumbar DRG as a different tissue type. Interestingly, despite comparable extraction yields and purities (Table 1), mouse DRG RIN values averaged 8.2 ± 0.3, whereas those from mouse skin were similar to the human samples, averaging 3.8 ± 0.7 (Fig. 1F).

**Table 1:**
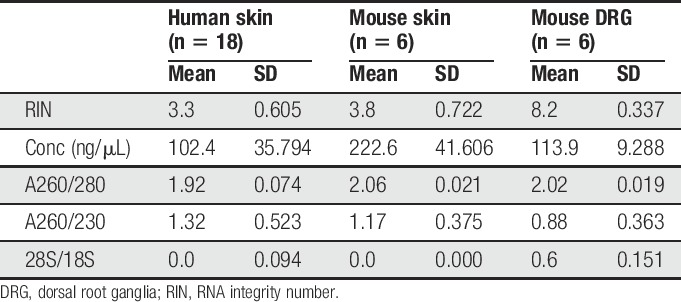
Evaluation indices for RNA extracted from skin or DRG samples with the RNA*later* + bead-milling protocol.

### 3.2. Snap freezing protects RNA integrity in dorsal root ganglia and spinal cord, but not skin

**Figure 2.**
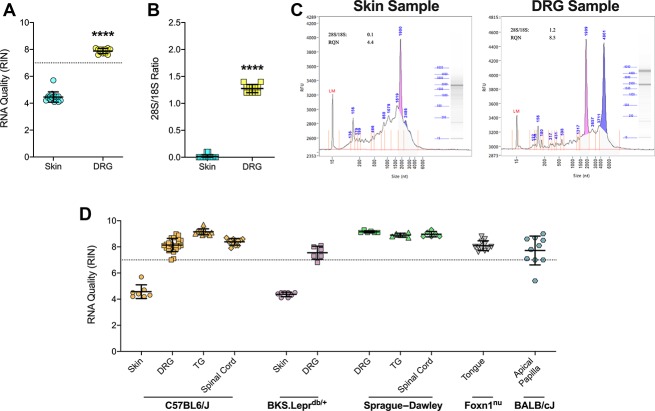
Snap freezing protects against RNA degradation in nervous tissues, but not skin. (A, B) Scatter plots of RIN values (A) and 28S/18S ratios (B) determined for extracted RNA from each snap-frozen, bead-milled mouse glabrous skin biopsy (yellow circles, n = 15) or lumbar DRG (yellow squares, n = 12). (C) Representative electropherograms with associated RIN and 28S/18S rRNA values for a mouse skin (left) and DRG sample (right). (D) Scatter plot of RIN values for glabrous skin (circles), lumbar DRG (squares), TG (triangles), lumbar spinal cord (diamonds), tongue (down triangles), and teeth (hexagons) samples collected from C57BL6/J mice (orange), C57BKS.Lepr^db/+^ mice (purple), Sprague–Dawley rats (green), Foxn1^nu^ mice (gray), or BALB/cJ mice (teal). Black horizontal bars in each plot represent the calculated group mean ± SD. ****P < 0.0001 by two-tailed Student's *t*-test. DRG, dorsal root ganglia; RIN, RNA integrity number; TG, trigeminal ganglia.

The failure of RNA*later* to preserve high-integrity RNA in both human and mouse skin biopsies required us to develop an alternative extraction protocol. Considering the nature of skin and its composition, it may be that the dense connective tissue restricts the perfusion of RNA*later*, thus thwarting chemical inactivation of ribonucleases.^23^ Therefore, we instead snap-froze samples immediately upon collection but extracted RNA using the same bead-milling approach. We again tested glabrous skin and lumbar DRG. Despite high RNA concentrations and purity (Table 2), acceptable RIN values were observed in DRG extracts only (Fig. 2A). Sample rRNA ratios mirrored these results (Fig. 2B), with skin samples exhibiting complete degradation of the 28S peak compared with DRG (Fig. 2C).

**Table 2:**
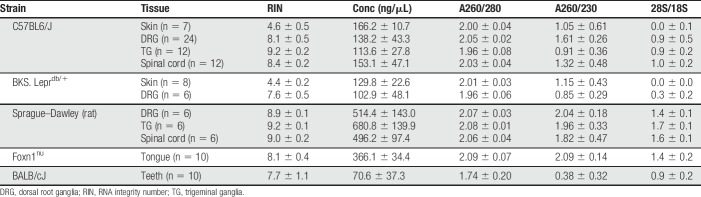
Evaluation indices for RNA extracted from various tissues with the snap-freeze + bead-milling protocol.

Despite the failure with skin, we continued testing this protocol with other tissue types collected from different mouse strains and Sprague–Dawley rats. As with DRG, this protocol extracted concentrated, pure, and intact RNA from virtually every sample (Fig. 2D and Table 3). These results were consistent across tissue types and across all rodent species and strains. We also confirmed the lack of any strain-dependent effect with this protocol on skin, given the low RIN values for glabrous skin from BKS.db/+ mice.

**Table 3:**
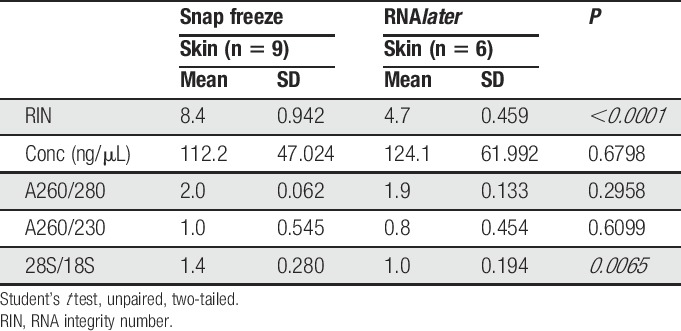
Evaluation indices for RNA extracted from mouse glabrous skin samples with either the snap-freeze or RNA*later* + cryosectioning protocol.

### 3.3. Cryosectioning prevents RNA degradation in snap-frozen skin samples

Although the snap-freeze/bead-mill protocol was effective with nonskin samples, we evaluated other extraction strategies since it did not preserve RNA in skin. Perhaps QIAzol, like RNA*later*, may not be diffusing through skin quickly enough to prevent ribonuclease-mediated RNA degradation. Thus, we considered to section skin with a cryostat instead of bead milling. In principle, endogenous ribonucleases should remain inactivated by the freezing temperature, and dicing the tissue into thin sections should promote rapid perfusion of QIAzol. Cryosectioning is used routinely to section tissue for laser capture microdissection, which permits the collection of laser-designated regions of tissue for RNA analysis.^29^ Moreover, cryosectioning has been shown effectively to extract high-quality RNA from notoriously difficult intervertebral disc tissue.^24^

**Figure 3.**
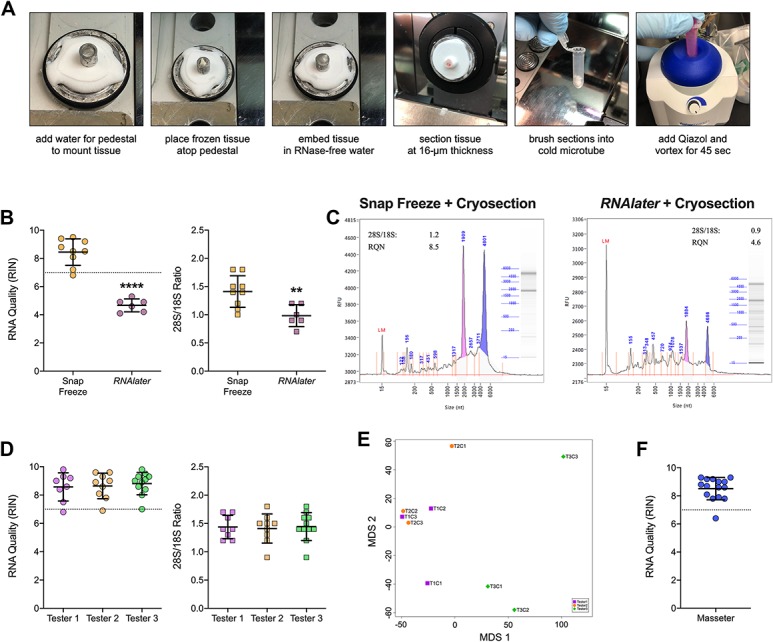
Extraction of high-integrity RNA from mouse skin requires both snap freezing and cryosectioning. (A) Images highlighting the preparation of a tissue sample for cryosectioning before RNA extraction and purification. (B) Scatter plots of RIN values and 28S/18S ratios determined for extracted RNA from mouse glabrous skin biopsies that were either snap frozen (orange circles, n = 9) or immersed in RNA*later* (purple circles, n = 6). (C) Representative electropherograms with associated RIN and 28S/18S rRNA values for a snap-frozen skin sample (left) and a skin sample immersed in RNA*later* (right) that both underwent cryosectioning before QIAzol extraction. (D) Scatter plots of RIN values (circles) and 28S/18S ratios (squares) determined for extracted RNA from snap-frozen mouse glabrous skin biopsies that were cryosectioned by either Tester 1 (purple, n = 8), Tester 2 (orange, n = 9), or Tester 3 (green, n = 11). (E) MDS plot of control mouse skin samples cryosectioned by Tester 1 (purple squares), Tester 2 (orange circles), and Tester 3 (green diamonds) to determine whether tester-specific batch effects occurred during extractions. (F) Scatter plot of RIN values determined for extracted RNA from mouse masseter muscle samples (blue circles) that were snap frozen and cryosectioned. Black horizontal bars in each plot represent the calculated group mean ± SD. ****P < 0.0001, **P < 0.01 by two-tailed Student's *t*-test. MDS, multidimensional scaling; RIN, RNA integrity number.

We adapted a similar approach as described previously.^4,24^ We acclimated a small metal cylinder in the cryostat and partially filled it with RNase-free water to form a pedestal (Fig. 3A). A skin sample was placed atop this pedestal and embedded with additional RNase-free water before mounting then cryosectioning. Collected sections were immersed in QIAzol, vortexed, and placed on ice before purification. We tested this cryosectioning approach on skin samples that were either snap-frozen or immersed in RNA*later*. All extracts yielded concentrated, pure RNA, although A260/230 values again were variable (Table 3). We subsequently determined that the cryosectioning protocol did in fact preserve high-integrity RNA in mouse skin samples, but only those that were snap frozen (Fig. 3B). With one exception, all snap-frozen samples exhibited RIN values ≥7.0, whereas samples immersed in RNA*later* were ≤5.5. rRNA ratios also were significantly higher in snap-frozen samples. Analysis of the electropherograms revealed that only snap freezing combined with cryosectioning prevented rRNA peak loss and accumulation of degraded RNA leftward of the 18S peak (Fig. 3C).

To test the reliability of this protocol, we had several laboratory members conduct the extraction on additional snap-frozen glabrous skin samples. The extraction protocol was first demonstrated to 3 different testers, and then each tester extracted samples on their own time. Each tester generated samples that had high RIN values and rRNA ratios (Fig. 3D and Table 4). Moreover, using sequencing results from control samples that were extracted by each tester, we observed no obvious batch effects. The multidimensional scaling plot showed that samples did not cluster, respectively, into 3 different groups by tester batch (Fig. 3E). In addition to skin, this protocol successfully extracted high-quality RNA from mouse masseter samples (Fig. 3F).

**Table 4:**
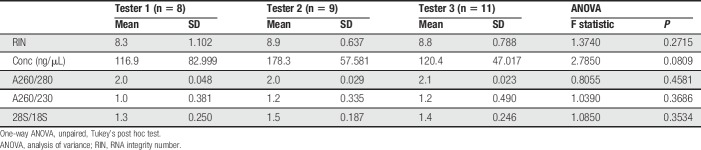
Evaluation indices for RNA extracted from mouse glabrous skin samples with the snap-freeze + cryosectioning protocol by different testers.

**Figure 4.**
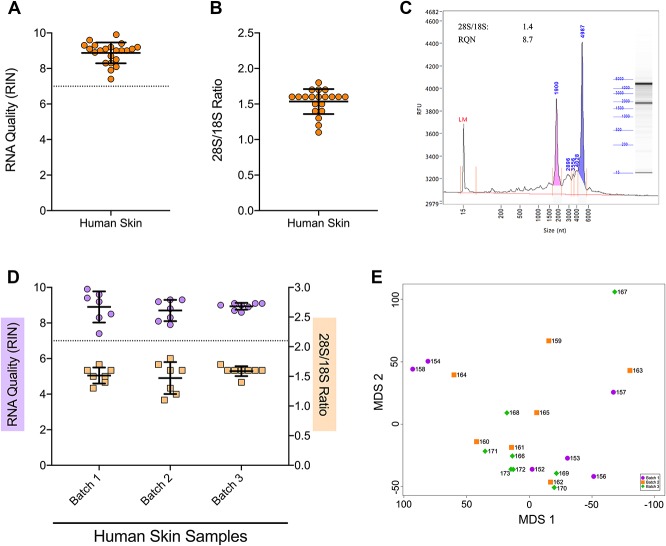
Cryosectioning preserves RNA integrity in patient skin biopsies. (A, B) Scatter plots of RIN values (A) and 28S/18S rRNA ratios (B) determined for extracted RNA from human hairy skin punch biopsies that were snap frozen on liquid nitrogen then cryosectioned (orange circles, n = 22). (C) Representative electropherogram with associated RIN and 28S/18S rRNA values from a snap-frozen, cryosectioned patient sample. (D) Scatter plots of RIN values (light purple circles, left *y*-axis) and 28S/18S ratios (light orange squares, right *y*-axis) determined for extracted RNA from snap-frozen human skin punch biopsies that were cryosectioned on one of 3 different processing days as batch 1 (n = 7), batch 2 (n = 7), or batch 3 (n = 8). (E) MDS plot of all human skin samples labeled according to processing batch 1 (purple circles), batch 2 (orange squares), or batch 3 (green diamonds) to determine whether batch-specific effects occurred during extraction. Each deidentification number corresponds to an individual patient sample. Black horizontal bars in each plot represent the calculated group mean ± SD. MDS, multidimensional scaling; RIN, RNA integrity number.

We proceeded to test human skin biopsies, given the consistent results with mouse skin. In contrast to the RNA*later/*bead-mill approach, snap freezing with cryosectioning extracted high-quality RNA from all samples (Fig. 4A, B and Table 5). Electropherograms consistently exhibited prominent rRNA peaks and minimal degraded RNA (Fig. 4C). We also checked for batch effects because these samples were processed on 3 different days. RNA integrity number values and rRNA ratios were consistently high for each day (Fig. 4D), and batch effects were not apparent since samples did not cluster into distinct groups by processing batch (Fig. 4E). Collectively, these data indicate that cryosectioning snap-frozen tissue is reliable and reproducible for the extraction of high-integrity RNA from skin as well as other tissue types such a muscle.

**Table 5:**
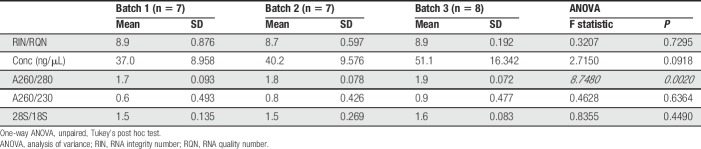
Evaluation indices for RNA extracted from different batches of human skin samples with the snap-freeze + cryosectioning protocol.

### 3.4. Partially degraded RNA can result in incorrect RNA integrity number value assignment

**Figure 5.**
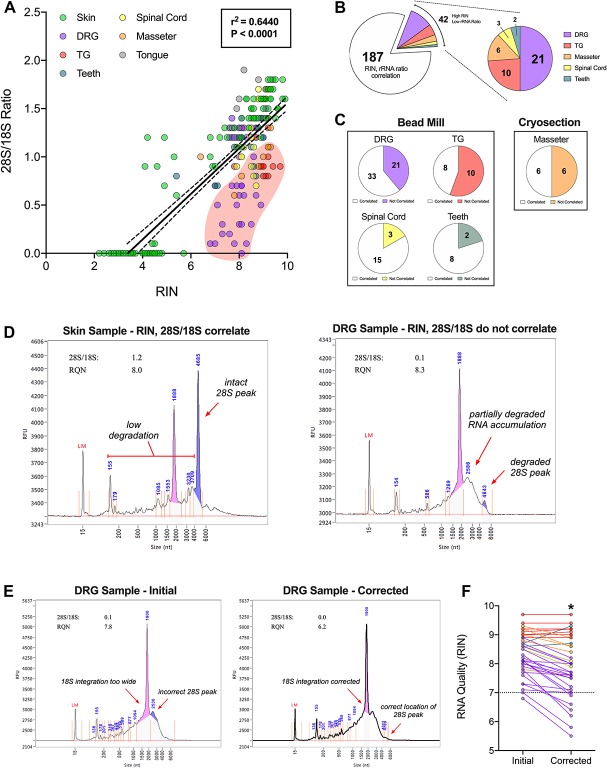
Electrophoretic anomalies can inflate RIN value assignment to extracted samples. (A) Correlation analysis between individual sample 28S/18S rRNA ratios and their respective assigned RIN values (Pearson, two-tailed, *P* < 0.0001) with corresponding linear regression curve (solid black line) with 95% confidence bands (dotted black lines). Various colors reflect the different tissue types, including skin (green), DRG (purple), TG (red), teeth (blue), spinal cord (yellow), masseter (orange), and tongue (gray). (B) Pie charts summarizing the number of samples with normal RIN and 28S/18S ratios (white, 187 samples) compared to samples with high RIN values but low 28S/18S rRNA ratios. Abnormal samples are further dissociated by tissue type with the values reflecting the number of samples per tissue type. (C) Pie charts highlighting the frequency of occurrence of abnormally high RIN values and low 28S/18S rRNA ratios (colored areas) compared to samples with acceptable RIN values and 28S/18S rRNA ratios. Tissue types were also grouped based on the method of tissue homogenization for RNA extraction, including bead milling (left) and cryosectioning (right). (D) Representative electropherograms from a skin sample (left) and a DRG sample (right) that highlight assignment of similar RIN values, despite differences in the 28S peak and accumulation of degraded RNA peaks (arrows). (E) Representative electropherograms from a DRG sample whereby initially misidentified peaks (left) were corrected (right), resulting in a profound reduction in the assigned RIN value. (F) Scatter plot highlighting changes to RIN value assignment after correction for all abnormal samples identified in (A). Colors correspond to those used above to differentiate the various tissue types. Colored lines connect the initial and corrected RINs for a single sample. *P < 0.05 by two-tailed Student's *t*-test. DRG, dorsal root ganglia; RIN, RNA integrity number; TG, trigeminal ganglia.

The 28S/18S ratio was the standard index of RNA integrity before development of the RIN algorithm.^35^ Although considered overly conservative, rRNA ratios generally correlate with sample RIN values. Interestingly, several samples exhibited high RIN values but low rRNA ratios. We investigated this further by plotting sample rRNA ratios as a function of their respective RIN values. Indeed, sample RIN values and rRNA ratios were positively correlated (Fig. 5A). Linear regression identified 42 samples with high RNA values but low rRNA ratios, including 21 DRG, 10 TG, 6 masseter, 3 spinal cord, and 2 teeth (Fig. 5B). Bead milling was used on 36 of these samples, whereas only 6 underwent cryosectioning (Fig. 5C).

We reassessed all 42 electropherograms and identified anomalies that were absent from other correlated samples. The most common anomalies included undetectable 28S peaks and RNA accumulation near the 18S peak. In one DRG electropherogram, the 28S peak is largely degraded while a broader peak accumulates rightward of the 18S peak. Despite the obvious degradation (28S/18S = 0.1), this particular sample received a higher RIN value of 8.3 compared to a skin sample with minimal degradation (28S/18S = 1.2) that received a RIN of 8.0 (Fig. 5D). These anomalies often caused the software to misidentify the 28S peak and overestimate the 18S peak width (Fig. 5E). Manual correction of these errors ultimately led to RIN reassignment, with most sample scores decreasing (Fig. 5F). Notably, RIN values for several samples dropped below the 7.0 cutoff after correction. We highlighted another DRG electropherogram that originally received a RIN value of 7.8 but was subsequently rescored to 6.2. Such anomalies must be investigated when encountered to avoid inclusion of unacceptable samples for quantitative mRNA sequencing.

## 4. Discussion

RNA is a labile biomolecule with pronounced susceptibility to chemical- and ribonuclease-mediated hydrolysis,^13^ necessitating great care during extraction to minimize the risk of degradation bias with quantitative DGE analysis. Although there is no consensus on the amount of RNA degradation permissible for DGE analysis, more studies are demonstrating the impact of degradation on sequencing data sets.^2,15,21,42^ Samples with poor RNA integrity have been shown to exhibit stochastic, disproportionate degradation of transcripts that inaccurately represented the active transcriptome.^15,45^ Degraded samples inevitably contain fragments that unevenly cover gene length, increasing intersample variance. Degradation also impacts signal/noise ratios, which are critical for subtracting background from sequencing reads.^28^ Altogether, these confounds increase false discovery rates as well as duplications.^25,38^ Alternative to poly(A)-enrichment used for mRNA-seq is ribodepletion for total RNA-seq, which can address some of these issues, especially with degraded samples, by specifically targeting rRNAs for removal. However, one major trade-off due to inclusion of nonpolyadenylated, intronic RNA is lower resolution of exonic reads, which consequently requires greater read depth and often more samples to achieve the same statistical power as mRNA-seq for DGE analysis.^22,39,47^ Still, degradation bias affects sequencing results regardless of enrichment approach; therefore, rigorous optimization of RNA extraction procedures can only benefit the accuracy of quantitative end applications such as DGE analysis.

Here, we developed a reliable protocol for high-integrity RNA extraction from skin samples intended for quantitative DGE analysis through unbiased mRNA-seq. We determined over the course of several iterations that extraction of high-integrity RNA from skin requires 2 steps: (1) immediate snap freezing upon collection and (2) homogenization through cryosectioning before extraction. Neither RNA-stabilizing solution nor bead milling preserved RNA integrity in skin. However, bead milling effectively extracted high-integrity RNA from other tissue types, including DRG, TG, spinal cord, tongue, and periapical lesions from teeth. During RNA integrity assessment, we observed subtle electrophoretic anomalies in ∼20% of samples that ultimately compromised RIN value assignment. Manually correcting for these anomalies typically reduced integrity scores, with several samples dropping below the designated cutoff. Collectively, these findings emphasize the need for pain researchers to rigorously optimize sample RNA extraction to accurately reflect gene expression changes used in DGE analysis.

Skin biopsies are an important resource, which includes their usefulness in clinical diagnostics and preclinical pain research.^16,37^ Utilization of quantitative mRNA-seq with skin holds much promise to better define the molecular bases of clinical pain conditions, including the contributions of keratinocytes and other non-neuronal cell types to peripheral sensitization.^1,21^ However, extracting intact RNA from skin has proven challenging.^44^ For our first iteration, we established that the primary obstacle in preserving RNA integrity in skin was the high ribonuclease expression, especially in epidermal keratinocytes undergoing terminal differentiation and cornification.^11^ After its failure, we deduced that RNA*later* was incompletely inhibiting the ribonucleases and thus switched to snap freezing. If snap freezing alone had been sufficient, sample RNA would have remained intact after bead milling. Our results clearly show this not to be the case, instead indicating that ribonuclease inhibition is lost somewhere before completing RNA extraction. Chemical inactivators could not maintain ribonuclease inhibition, as neither immersion in RNA*later* at collection or in QIAzol after snap freezing–preserved RNA integrity. Similar ineffectiveness was also reported with articular cartilage.^23^ Collectively, these indicated that a second major obstacle, the dense, interwoven connective tissue, impedes rapid chemical inactivation of skin ribonucleases. Thus, we needed an approach that maintained the cold-induced inactivation of ribonucleases while simultaneously permitting mechanical breakdown of skin. Remarkably, cryostat sectioning of skin biopsies was sufficient to preserve RNA integrity through extraction with QIAzol. Although slightly more time consuming, this method is easy to learn and inexpensive. Plus, cryostats are virtually ubiquitous in biomedical research institutions.

As noted previously,^19^ we observed a significant positive correlation between RIN values and rRNA ratios from all samples tested in this study. Interestingly, we identified 42 samples that oddly exhibited high RIN values yet low rRNA ratios. None of the 60 snap-frozen skin samples that underwent cryosectioning were among these abnormal samples. Rather, ∼90% were samples homogenized with the Bead Ruptor, suggesting that bead milling risks additional RNA degradation. Despite the potential of mechanically shearing transcripts and heat-induced oligomerization,^3,31^ the majority of bead-milled samples (∼70%) still exhibited normal RIN values and rRNA ratios. Alternatively, these anomalies may have arisen as a consequence of extended dissection. Most of the abnormal samples were in fact DRG, which are collected through a notoriously slow dissection process. We determined through re-examination of the electropherograms that the automated software frequently, but not always, misidentified the 28S peak and overestimated the 18S peak width. We communicated these findings to Agilent technical staff who also guided our correction approach. Of the 42 manually corrected electropherograms, 30 were reassigned lower RIN values, with 6 samples falling below the cutoff. Careful evaluation of the electropherograms at this stage is critical to identify problematic samples before sequencing.

In this study, we used RIN value assignment as the primary metric for RNA integrity and deemed RIN values ≥7.0 as acceptable for mRNA-seq.^13,15,18,35^ Despite its status as the current standard metric, we do recognize the growing claim that RIN values may not adequately reflect sample mRNA integrity. This is largely based on the premise that the RIN algorithm heavily weighs rRNA integrity, which recently was shown only to weakly associate with sample transcript quality.^20^ Considering tissue-specific differential RNA degradation and its impact on DGE analysis, alternative transcript integrity metrics, such as transcript integrity number^45^ or quality surrogate variable analysis,^20^ should also be considered for future sequencing efforts.

We developed and validated a robust extraction protocol that combines snap freezing with cryosectioning to routinely yield high-integrity RNA from rodent and human skin biopsies. We also validated RNA extraction protocols for other tissues commonly used in the pain field. Nevertheless, pain researchers must rigorously validate their RNA extraction protocols and abide by strict quality control standards. We also advocate for better reporting standards whereby authors detail methodological information and all data pertaining to their RNA-seq workflows, the extraction approach and sample RINs notwithstanding.^36^ Through adherence to meticulous RNA-processing standards, especially with patient samples, quantitative DGE analysis will be an extremely powerful approach that advances new discoveries in pain research.

## Disclosures

The authors have no conflicts of interest to declare.

This study was supported by NIH grants: T32DE14318 (K.M.H, P.M.L.), F32DK118841 (P.M.L.), UTHSCSA Endodontics Departmental Funds, and the USAA President's Council Endowment. RNA integrity data were generated in the Genome Sequencing Facility, which is supported by UT Health San Antonio, NIH-NCI P30CA054174 (Cancer Center at UT Health San Antonio), NIH 1S10OD021805-01 (Shared Instrument grant), and CPRIT Core Facility Award (RP160732).
